# Stereochemical identification of glucans by oligothiophenes enables cellulose anatomical mapping in plant tissues

**DOI:** 10.1038/s41598-018-21466-y

**Published:** 2018-02-15

**Authors:** Ferdinand X. Choong, Marcus Bäck, Anette Schulz, K. Peter. R. Nilsson, Ulrica Edlund, Agneta Richter-Dahlfors

**Affiliations:** 1 Swedish Medical Nanoscience Center, Department of Neuroscience, Karolinska Institutet, Stockholm, SE-171 77 Sweden; 2 Department of Chemistry, IFM, Linköping University, Linköping, SE-581 83 Sweden; 3 Fibre and Polymer Technology, KTH Royal Institute of Technology, Stockholm, SE-100 44 Sweden

## Abstract

Efficient use of plant-derived materials requires enabling technologies for non-disruptive composition analysis. The ability to identify and spatially locate polysaccharides in native plant tissues is difficult but essential. Here, we develop an optical method for cellulose identification using the structure-responsive, heptameric oligothiophene h-FTAA as molecular fluorophore. Spectrophotometric analysis of h-FTAA interacting with closely related glucans revealed an exceptional specificity for β-linked glucans. This optical, non-disruptive method for stereochemical differentiation of glycosidic linkages was next used for *in situ* composition analysis in plants. Multi-laser/multi-detector analysis developed herein revealed spatial localization of cellulose and structural cell wall features such as plasmodesmata and perforated sieve plates of the phloem. Simultaneous imaging of intrinsically fluorescent components revealed the spatial relationship between cell walls and other organelles, such as chloroplasts and lignified annular thickenings of the trachea, with precision at the sub-cellular scale. Our non-destructive method for cellulose identification lays the foundation for the emergence of anatomical maps of the chemical constituents in plant tissues. This rapid and versatile method will likely benefit the plant science research fields and may serve the biorefinery industry as reporter for feedstock optimization as well as in-line monitoring of cellulose reactions during standard operations.

## Introduction

Composition analysis of polysaccharide rich materials addresses the need for non-destructive, accurate and efficient techniques, preferentially at low cost. Oligothiophenes is a class of structure-responsive, fluorescent molecules that recently were shown to serve as highly sensitive optical sensors of lignocellulosic materials^1,2^. Upon binding to polysaccharides via non-covalent interactions, geometric changes are induced in the thiophene backbones, which results in an ON/OFF like switching of fluorescence activation. Unique spectral signatures are thereby produced, which can be used to identify the specific biomolecular targets^3–8^. Whereas most polysaccharides are invisible to standard immunofluorescence techniques^9^ as they lack unique surface epitopes, the newly reported oligothiophene-based method enables optical analysis by spectrophotometry as well as spectral imaging^1,2^. This technique is thus highly advantageous for visualization of repetitive polysaccharide structures.

Glucose-based polysaccharides, also known as glucans, show great variation in stereochemistry and degree of polymerization. The structural and stereochemical variability, which provides an impressive range of chemical and physical properties to the large number of glucans, makes polysaccharides challenging to analyze^10–12^. Further complexity is added when identifying and differentiating glucans in mixtures^9,11^. The standard techniques for carbohydrate determination are mainly disruptive in the sense that the biomass is degraded prior to, or during analysis^13,14^. Furthermore, mainstay methods, such as ion exchange chromatography, identify carbohydrates based on their constituent monosaccharides. As a result, such methods cannot provide selectivity when applied to homopolysaccharides and fails accordingly to differentiate amylose from cellulose in mixed samples.

The oligothiophene-based technology, based on pentameric and heptameric oligothiophenes, has shown exceptional potential to specifically detect the structure and purity of cellulose in lignocellulose and microbial biomasses^1,2^. Binding of the oligothiophene molecules to cellulose polysaccharides involves flattening of the thiophene backbone^3,4,15^. This geometric change in the bound oligothiophene is associated with a red shift in the excitation maximum (λ_max_) and increased fluorescence emission, as observed when recording spectra between 350–750 nm^1,2^. In addition, the molecules enable monitoring of dynamic processes, such as enzymatic degradation of cellulose by cellulases, as well as the formation of cellulose in growing *Salmonella* biofilms^2^.

**Figure 1:**
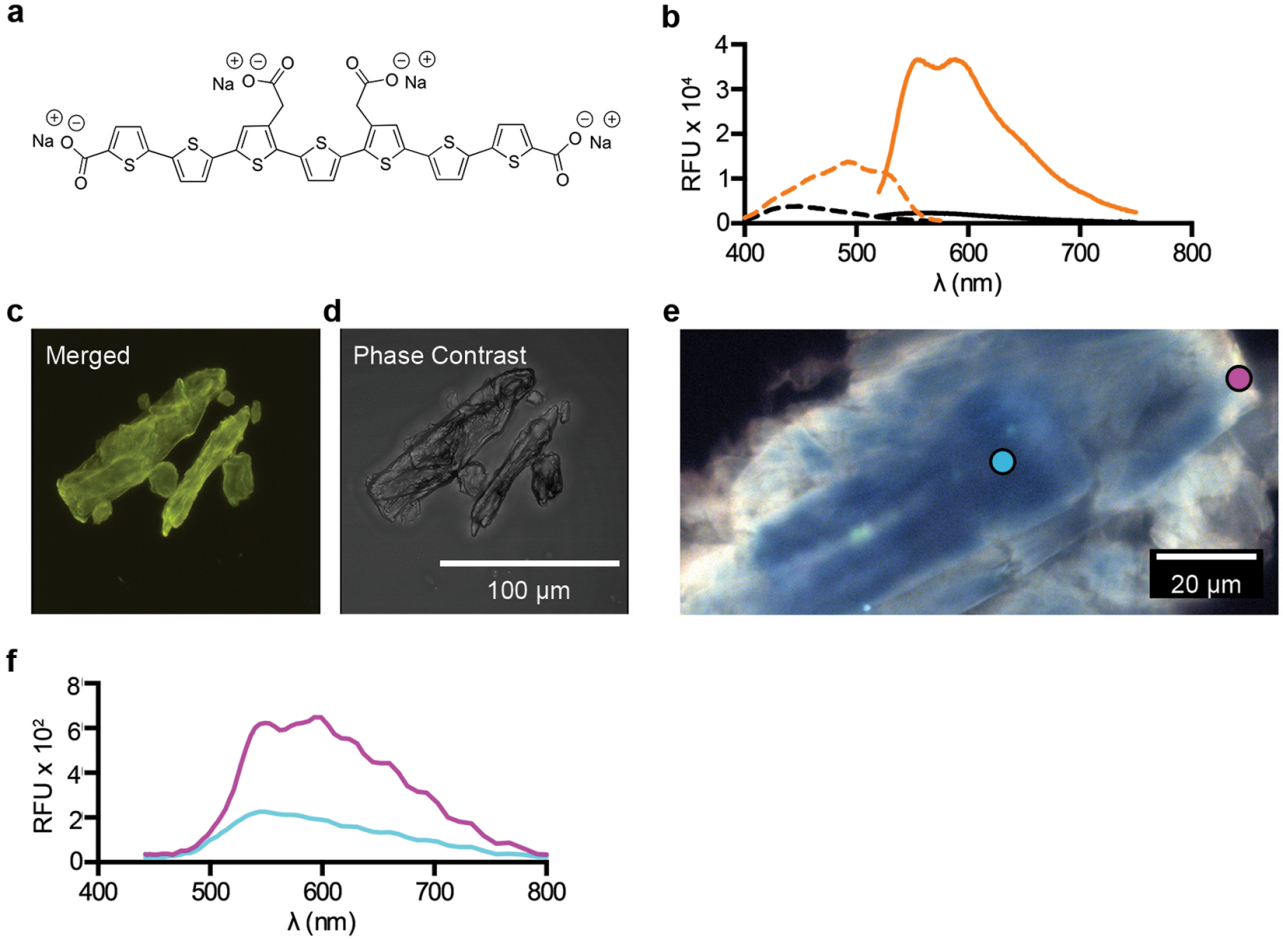
Optical characteristics of h-FTAA bound to cellulose. (**a**) The chemical structure of h-FTAA. (**b**) Spec-Plot showing the excitation (dashed line) and emission (solid line) spectra of h-FTAA in unbound form (black) and when bound to M. cellulose (orange). Average data complied from 3 independent experiments are shown. (**c**) Multi-detector analysis using CLSM, showing a 3D brightest point projection of an image stack (24.96 μm stack, z-step = 0.64 μm) of h-FTAA bound to M. cellulose. Excitation by a 473 nm laser is detected by PMTs between 490–540 nm (green), 575–620 nm (yellow) and 655–755 nm (red) and merged into one image. (**d**) The crystal morphology of (**c**) shown by phase contrast. (**e**) Spectral imaging of h-FTAA bound to M. cellulose viewed under a fluorescence microscope with a 436 nm excitation laser and a spectral detector module. The crystal’s inner region (cyan circle) and edge (magenta circle) are indicated. (**f**) Spec-Plot showing the emission spectra of h-FTAA bound at the cellulose crystal’s inner region (cyan) and edge (magenta). Spectra originate from analysis of 4 pixels in each of the inner region and edge in fluorescence micrographs.

Here, we address the underlying molecular details of the ability of oligothiophenes to serve as optical sensors for cellulose. Using pure extracts of cellulose, well-defined cellodextrins and a range of glucans (Supplementary Table 1), we perform detailed analyses of structural determinants promoting binding of the oligothiophene h-FTAA^2,15,16^ (Fig. 1a). By studying the optical changes h-FTAA undergoes as it transit from unbound to bound states, we explore a variety of methods for glucan detection in pure extracts, thereby laying the foundation for analysis of samples of higher complexity. Using potato and onion as model organisms, we demonstrate *in situ* staining of cellulose in its native state as a nanocomposite in plants. This demonstrates a novel non-destructive method to visualize the anatomical location of cellulose in plant tissues by standard fluorescence microscopy.

## Results

### Optotracing of microcrystalline cellulose fibrils

To define the optical changes h-FTAA undergoes when binding to cellulose, we first defined the spectra of unbound probe in phosphate-buffered saline (PBS) using spectrophotometric recordings between 400–750 nm. When plotting the relative fluorescence units (RFU) in a spectral plot (Spec-Plot), an excitation λ_max_ of 446.5 ± 12.3 nm and emission λ_max_ of 564.5 ± 19.8 nm were observed, despite comparatively low RFU (Fig. 1b). We then analysed h-FTAA mixed with commercially available microcrystalline cellulose fibrils (M. cellulose). The Spec-Plot showed that binding of h-FTAA to M. cellulose brings a red shift in the excitation λ_max_ to 497.5 ± 17.5 nm and the appearance of characteristic shoulders at 460 nm and 525 nm. A unique emission spectrum was also observed, with dual λ_max_ at 558.5 ± 10.8 nm and 593 ± 9.38 nm. Single wavelength excitation at 500 nm showed that binding of h-FTAA to M. cellulose resulted in 14.4-fold increased fluorescence across the entire emission spectrum compared to the signal from the unbound oligothiophene.

To enable fluorescence visualization of the cellulose fibrils, we next translated the defined Spec-Plot signatures to optical settings in a confocal laser scanning microscope (CLSM) and performed multi-detector imaging. After positioning a sample of h-FTAA mixed with M. cellulose on a microscope slide, we used the CLSMs pre-set 473 nm laser line to excite the sample, while detecting fluorescence by the three photomultiplier tubes (PMTs) at 490–540 nm (green), 575–620 nm (yellow) and 655–755 nm (red) (Supplementary Fig. 1a). Image stacks (circa 25 μm stacks collected with 0.64 μm z-step between focal planes) from each PMT were converted to 3D projections and pseudo-coloured to the visible spectrum (Supplementary Fig. 1b–d). Merging the 3D projections from the PMTs resulted in yellow fluorescence from the M. cellulose crystal (Fig. 1c). The crystal morphology corresponded excellently to the structure observed in the image collected under phase-contrast (Fig. 1d). This indicated extensive binding of h-FTAA throughout the entirety of the cellulose crystal.

To investigate whether different types of binding sites for h-FTAA are present along the M. cellulose crystal, we utilised the well-known fact that binding of h-FTAA induces structural changes in the oligothiophene backbone and the associated changes in spectral profiles serve as identifiers of the bound targets^1–4,15,16^. To screen all possible geometries h-FTAA undertakes when binding to cellulose, a sample of h-FTAA mixed with M. cellulose was positioned on a microscope slide, excited at 436 nm and analysed under a fluorescence microscope equipped with a SpectraCube module. This spectral camera automatically applies pseudo-colouration as a function of the wavelength and intensity of emitted fluorescence. Intense fluorescence from h-FTAA was found permeating throughout the entire M. cellulose crystal, with two distinct pseudo-coloured regions (Fig. 1e). White fluorescence was observed at the edge of the crystal, whereas blue fluorescence dominated in all remaining regions. When analysing the emission spectra in pixels at the two regions, the Spec-Plot showed emission λ_max_ 590 ± 31.3 nm at the crystal edge and 556 ± 16.4 nm at remaining blue fluorescent regions (Fig. 1f). This implies that h-FTAA bound at the crystal edge adopt a more linearized structure compared to molecules bound at other sites. To test this hypothesis, we performed multi-laser/multi-detector imaging, which utilizes the multiple lasers and detectors that are present on basic CLSM systems (Supplementary Fig. 1e). Optical sectioning of the sample using consecutive excitation at 405 nm and 473 nm showed pseudo-coloured magenta fluorescence exclusively at the crystal edge, whereas other regions of the crystal showed a homogenous, pseudo-coloured cyan fluorescence (Supplementary Fig. 1f). These fluorescence signals were absent in unstained M. cellulose crystals (Supplementary Fig. 1g). The differential colouration suggests the presence of at least two different types of binding sites for h-FTAA on M. cellulose. Furthermore, the excellent correlation of data obtained by spectrophotometric recording, spectral imaging and CLSM shows that h-FTAA reporting of M. cellulose can be performed on a range of analytical platforms, which provides great versatility to the method.

### h-FTAA specifically binds to and linearizes against the polysaccharide backbone of cellulose

**Figure 2:**
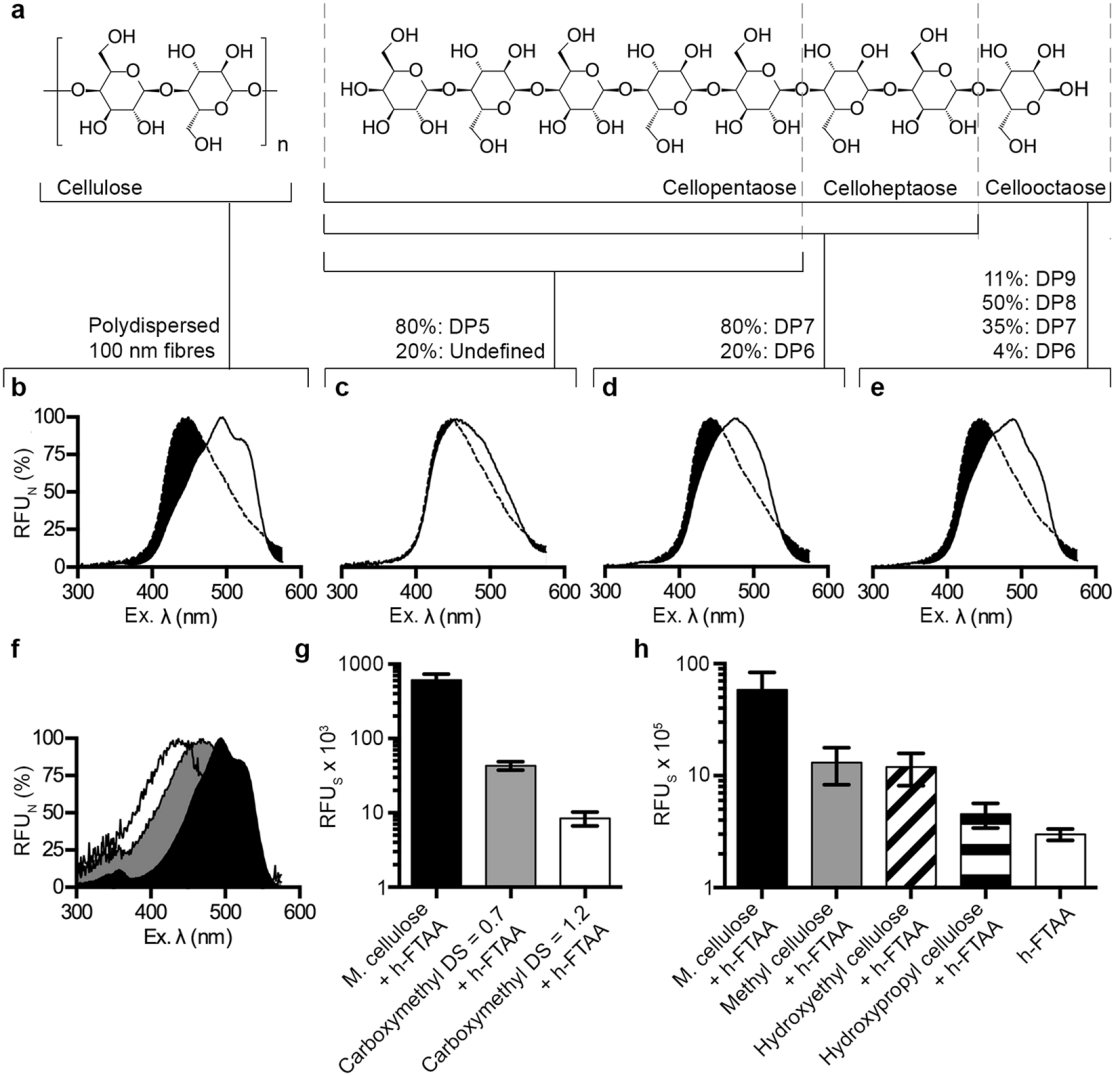
h-FTAA binds the β(1–4) linked oligoglucopyranosyl chain and requires a minimum length for cellulose specific linearization. (**a**) Chemical structure of cellulose and oligosaccharides demarcated at penta-, hepta- and octameric lengths. The composition of each polydispersed sample as specified by the manufacturer is shown. (**b**–**e**) Normalized Spec-Plot showing the excitation spectra of h-FTAA interacting with (**b**) M. cellulose, (**c**) cellopentaose, (**d**) celloheptaose and (**e**) cellooctaose. Normalisation is performed by plotting the emitted fluorescence at each excitation wavelength as percentage of the highest and lowest values in the spectrum. (**f**) Normalized Spec-Plot showing the excitation spectra of h-FTAA interacting with M. cellulose (black), carboxymethyl cellulose with a degree of substitution (DS) of 0.7 (grey) and carboxymethyl cellulose, DS 1.2 (white). (**g**) Emitted fluorescence (RFU_S_) of h-FTAA excited at 500 nm when interacting with M. cellulose and carboxymethyl celluloses with DS 0.7 and 1.2. (**h**) Emitted fluorescence (RFU_S_) of h-FTAA interacting with M. cellulose, methyl cellulose, hydroxyethyl cellulose and hydroxypropyl cellulose when excited at 500 nm. h-FTAA in unbound form is used as comparator. Average data from 3 independent experiments are shown in (**b**–**h**), error bars in (**g**,**h**) = standard deviation.

While binding of oligothiophenes to M. cellulose has been previously reported^1^, the molecular details of interaction are not known. To determine the minimal length required for binding, the fluorescence signatures from h-FTAA bound to cellulose oligosaccharides of defined lengths were analysed (Fig. 2a, Supplementary Table 1). To enable easy comparison of wavelengths representing the peak amplitudes, we normalised the emitted fluorescence (RFU_N_) by calculating the percentage of the emitted fluorescence at each excitation wavelength relative to the highest and lowest values in the spectrum. The RFU_N_ was then plotted in Normalised Spec-Plots (Fig. 2b–e). As positive comparator, we used the optical signature from h-FTAA bound to M. cellulose. The Normalised Spec-Plots showed the typical shift of excitation λ_max_ from 447 ± 12.0 nm of unbound oligothiophene to 497.5 ± 17.5 nm, as well as the appearance of the two typical shoulders at 460 and 525 nm (Fig. 2b). When adding h-FTAA to cellopentaose (5 glucose residues, degree of polymerization (DP) of 5), a small shift in excitation λ_max_ to 469.5 ± 26.7 nm was observed compared to the unbound oligothiophenes (Fig. 2c). By increasing the oligosaccharide length to DP 7, celloheptaose, a bell-shaped excitation spectrum from bound h-FTAA was observed with excitation λ_max_ at 474 ± 17.8 nm (Fig. 2d). While this optical signature is more similar to the signal obtained by h-FTAA bound to M. cellulose, the distinct shoulders were lacking. Further increase of the oligosaccharide length to DP 8, cellooctaose, resulted in a distinctly red shifted excitation λ_max_ at 485.5 ± 32.5 nm, as well as the appearance of the shoulders at 460 and 525 nm (Fig. 2e). The optical signature of h-FTAA bound to cellooctacose thus mimics the signature from h-FTAA bound to M. cellulose. Careful visual inspection revealed, however, differences between the spectra. These were quantified by calculating the ratio between the peak RFU at 525 nm (the shoulder) and at 498 nm (the excitation λ_max_) in each spectrum. h-FTAA bound to cellooctaose showed a peak ratio of 0.695, whereas binding to M. cellulose generated a peak ratio of 0.852. This suggests that a proportion of the h-FTAA population bound cellooctaose in a conformation that gives the typical signature of cellulose binding, whereas the remaining part of the population adopts other conformations. This result is in accordance with the heterogeneous composition of the cellooctaose preparation used. Whereas it predominantly consists of cellooctaose (50%), it also contains 11% DP9, 35% DP7 and 4% DP6. Collectively, these experiments show that h-FTAA requires a minimum of 8 DP for proper cellulose binding.

Binding of h-FTAA to cellulose may occur via direct alignment to the β (1 → 4) D-glucopyranosyl chains, along the face of the glucopyranose rings. If so, the addition of chemical motifs to -OH groups projecting above and below the polysaccharide backbone is likely to obstruct h-FTAA binding. To test this hypothesis, we applied h-FTAA to carboxymethyl celluloses with different degrees of substitution (DS) and profiled the interaction by spectrophotometry. The Normalised Spec-Plot showed that compared to the excitation λ_max_ of 508.5 ± 15.7 nm of h-FTAA bound to M. cellulose, minimal substitution of outward projecting H atoms at a ratio of 0.7 carboxymethyl groups per anhydroglucose unit resulted in excitation λ_max_ of 470 ± 11.4 nm (Fig. 2f). Further carboxymethyl substitution to a ratio of 1.2 groups per anhydroglucose unit showed an excitation λ_max_ of 433 ± 17.7 nm. The shifts in excitation λ_max_ were accompanied by a 10- and 100-fold reduction of emission for h-FTAA bound to carboxymethylated cellulose (DS 0.7 and DS 1.2, respectively), as compared to h-FTAA bound to M. cellulose (Fig. 2g). The presence of carboxymethyl groups thus hinders binding of h-FTAA to M. cellulose.

When expanding our analysis to cellulose ethers with methyl or hydroxyethyl groups, we observed circa 10-fold reduction of emission intensities from bound h-FTAA compared to the signal obtained when bound to M. cellulose (Fig. 2h). Interestingly, emission from h-FTAA bound to hydroxypropylated cellulose was indistinguishable from the background signal of unbound h-FTAA. Collectively, these data suggest that any modification of the cellulose polysaccharide significantly affects the binding of the oligothiophene. R-groups present on the polysaccharide chain appear to alter molecular surface geographies, effectively altering the interactions with h-FTAA.

### Stereochemical selectivity of h-FTAA for β-linked glucans

**Figure 3:**
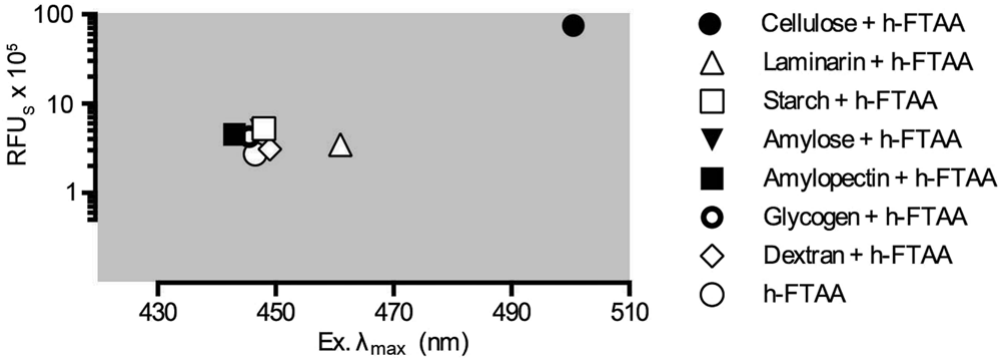
Stereochemical differentiation of α and β configurated glucopyranosyl units by h-FTAA. Cluster analysis shows emitted fluorescence (RFU_S_) plotted against excitation λ_max_ of h-FTAA in PBS and of h-FTAA interacting with α configurated starch, amylose, amylopectin, glycogen and dextran as well as with β configurated laminarin and M. cellulose. Averages from 3 independent experiments for each condition are shown.

Observing the exceptional sensitivity of h-FTAA to minute changes in polysaccharide lengths and chemical modifications, we next analysed whether optical recording of h-FTAA interacting with glucans can be applied as a novel method to detect stereochemical differences in glycosidic linkages. We therefore mixed h-FTAA with M. cellulose, laminarin, starch, amylose, amylopectin, glycogen and dextran (Supplementary Table 2), collected excitation spectra of each mixture and identified the wavelengths representing peak amplitudes using Normalised Spec-Plots (Supplementary Fig. 2a–h). Analysis of the emitted fluorescence from each sample excited at 500 nm, known to induce maximum emission from h-FTAA bound to cellulose, showed distinctly lower RFU_S_ for the tested glucans compared to cellulose (Supplementary Fig. 2i). Plotting RFU_S_ versus excitation λ_max_ from each individual spectrum, the striking difference was made apparent. All glucans with α configurated glucopyranosyl units formed a tight cluster distinctly overlapping the signal from unbound h-FTAA (Fig. 3). In contrast, binding to laminarin, a glucan with primarily β(1 → 3) and β(1 → 6) configuration, resulted in +15 nm change in excitation λ_max_ and 1.9-fold increase in RFU_S_. The optical shift was further enhanced for h-FTAA bound to cellulose showing a red shift of +54 nm in excitation λ_max_ and 27.5-fold increase in RFU_S_ compared to the unbound h-FTAA. Collectively, this demonstrates a very high selectivity of h-FTAA for β glycosidic bonds. To determine if this selectivity was influenced by the insolubility or the crystalline morphology of M. cellulose, h-FTAA was mixed with samples of cellulose nanofibrils and amorphous cellulose. The excitation spectrum of each sample was collected and analysed on Normalised Spec-Plots (Supplementary Figure 2j,k). Analysis showed that the optical signature of h-FTAA bound to cellulose nanofibrils and amorphous cellulose were identical to that of h-FTAA binding to M. cellulose (Supplementary Figure 2a), with excitation λ_max_ at 508.5 nm flanked by accessory peaks. This indicated that the cellulose optical signature of h-FTAA is independent of the solubility and morphology of cellulose. Optical recordings of h-FTAA may therefore be applied as a novel, rapid, non-disruptive method to determine the stereochemistry of glycosidic linkages in glucans.

### h-FTAA enables anatomical mapping of cellulose in plant tissues

**Figure 4:**
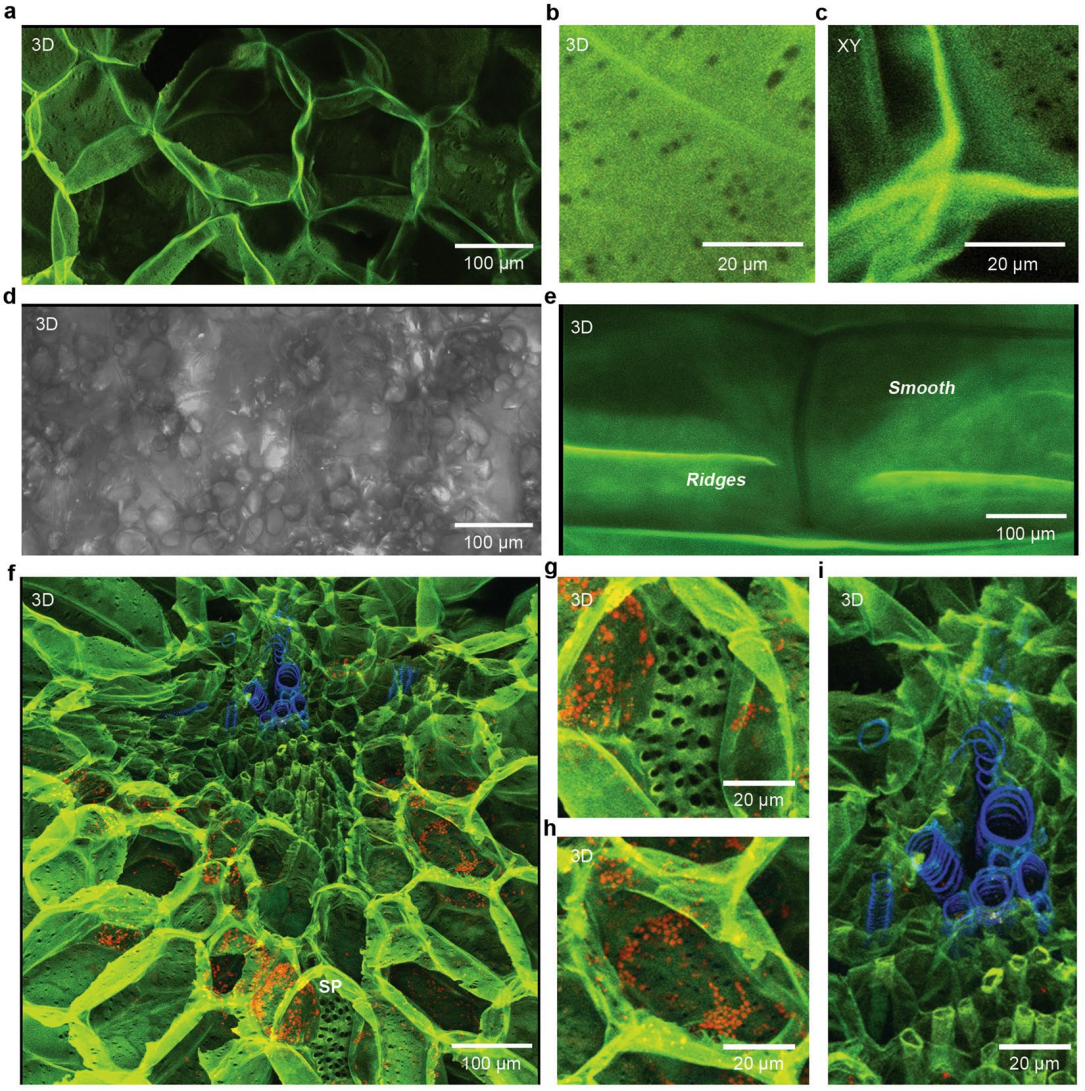
Multi-laser/multi-detector analysis enables cellulose anatomical mapping in plant tissues. (**a**–**c**) Multi-laser/multi-detector analysis performed by sequential excitation with 405 nm, 473 nm, 559 nm and 635 nm lasers and frame-wise detection by PMTs at 430–455 nm (blue), 490–540 nm (green), 575–620 nm (yellow) and 655–755 nm (red), respectively. (**a**) A 3D brightest point projection of an image stack (92.72 μm stack, z-step = 1.22 μm) of potato parenchyma stained with h-FTAA showing cellulose (green/yellow) located to the cell walls. (**b** and **c**) Structures in the cell walls visualised by h-FTAA staining of potato parenchyma. (**b**) 3D brightest point projection of an image stack (58.8 μm stack, z-step = 0.56 μm) shows single and paired plasmodesmata. (**c**) Single optical section from the stack in (**b**), showing intercellular junctions. (**d**) Phase contrast image of the image stack shown in (**a**). (**e**) Multi-laser/multi-detector analysis of onion epithelium stained with h-FTAA. A 3D brightest point projection of an image stack (38.64 μm stack, z-step = 0.56 μm) shows well-delineated onion cells with cellulose in green/yellow. Smooth areas as well as ridges in the cell walls are indicated. (**f**) Multi-laser/multi-detector analysis of the vascular bundle of onion scale stained with h-FTAA. A 3D brightest point projection of an image stack (85.4 μm stack, z-step = 1.22 μm) shows a cross-section of the vascular bundle, with cell walls clearly outlined by h-FTAA bound to cellulose (green/yellow). SP = sieve plates (**g**–**i**) Enlarged areas from (**f**) show specialised structures, such as (**g**) perforated sieve plates of the phloem. (**h**) Chlorophyll (red) appearing in distinct intracellular compartments is visualised based on intrinsic fluorescence. (**i**) Fluorescent lignin (blue) is observed in the cell wall thickenings of the trachea in the vascular bundle. Readers are referred to an animation of this image stack (Supplementary Movie 1), which identifies cell wall thickenings to be of spiral type.

Having demonstrated the exceptional specificity of h-FTAA in reporting cellulose, we next analysed whether this high quantum yield molecule can be used to visually map the anatomical location of cellulose in plant tissues. We therefore applied CLSM-based multi-laser/multi-detector analysis of thin tissue sections of potato stained with h-FTAA. Sequential imaging of this tissue at 405/430–450 nm, 473/490–540 nm, 535/575–620 nm and 635/655–755 nm, generated image stacks which we analysed by 3D projections. Large multi-faced cell walls of medullary parenchyma cells were observed, whose green-yellow fluorescence, originating from excitation at 473 nm and 535 nm, implies cellulose as the principle component (Fig. 4a). Consistent with the cellulose specificity, no intracellular organelles were observed. Cells thus appeared as hollow honeycomb structures within the tissue. By applying higher magnification, intricate details on the inner face of cell walls were observed. Numerous single and paired plasmodesmata of varying sizes were observed in random distribution (Fig. 4b). Intercellular spaces could also be visualised in detail at the junction of adjacent cell walls (Fig. 4c). These detailed microscope images are in stark contrast to information gained from 3D projections of image stacks collected from the same tissue under phase contrast, showing cell walls as transparent and indistinct and intracellular compartment seemingly filled with granules (Fig. 4d, Supplementary Fig. 3a).

To test whether this staining technique is generally applicable to plant tissues, we stained a single layer of onion epithelium. Multi-laser/multi-detector analysis of tissue stained with h-FTAA generated image stacks, whose corresponding 3D-projections showed a mixture of smooth surfaces and jagged ridges on the cell walls of the continuous cell layer (Fig. 4e, Supplementary Fig. 3b). The vascular bundle of onion scales has a more complex anatomy compared to the epithelium. We therefore repeated the multi-laser/multi-detector analysis of tissue stained with h-FTAA, this time imaging the vascular bundle. The 3D-projections showed an obvious localisation of cellulose to the cell walls, as assessed by the optical signal from bound h-FTAA when excited at 473 and 535 nm (Fig. 4f, Supplementary Fig. 3c,d). In contrast to the epithelial cell morphology, a larger variety of sizes were observed among the vascular bundle cells. Perforated sieve plates of the phloem could also be visualised and their pores became even more apparent when the image was analysed at higher magnification (Fig. 4g).

Since our multi-laser/multi-detector method distinctly separates fluorescence signals, we analysed whether intrinsic tissue fluorescence can be used to our advantage, to provide further details on the anatomical location of chemical constituents in plants. The green pigment chlorophyll, a molecule essential for plant photosynthesis, is known to fluoresce when excited at 440 nm and 660 nm^17,18^. We therefore excited the h-FTAA stained onion scale at 635 nm, while performing multi-laser/multi-detector analysis on the region containing the vascular bundle. Chlorophyll was clearly identified in distinct intracellular compartments within cells surrounded by h-FTAA stained, cellulose-containing cell walls (Fig. 4h). The intracellular compartments are most likely chloroplasts, since this organelle is characterised by its high concentration of chlorophyll^17^. Lignin is another plant constituent that fluoresce when excited by UV and visible light^19–23^. By applying the multi-laser/multi-detector analysis, lignin located to trachea cell wall thickenings in the vascular bundle was observed. With the exceptional optical resolution of CLSM and the possibility provided by this method to analyse thick tissue sections, we could define the type of thickening as spiral, rather than annual or reticulate (Fig. 4i, Supplementary Video 1).

Collectively, our microscopy-based experiments demonstrate that multi-laser/multi-detector analysis of h-FTAA stained tissues enables distinct anatomical mapping of cellulose to cell walls in plant tissues. The fluorescence-based method also allows identification of the spatial relationship with intrinsically fluorescent organelles. Detailed structures become even more evident when studying an animation of the 3D-projection of the tissue (Supplementary Video 2). As this non-destructive method requires minimal sample preparation, interference with the tissue architecture is kept to a minimum, thereby providing accuracy to the method at the sub-cellular scale.

## Discussion

Oligothiophenes constitute a novel class of molecular fluorophores for the detection and visualisation of cellulose. Having previously shown the sensitivity of oligothiophenes of a specific chemistry for cellulose^1,2^, we further demonstrated here the molecular details of the interaction. We determined that the characteristic cellulose signature is due to the conjugation of the heptameric oligothiophenes to a minimum of 8 β(1 → 4) linked glucose monomers. This interaction provides resolution at the molecular level and easily discriminates cellulose from highly similar stereoisomers and other glucans. The specificity for cellulose was maintained despite the complexity of native plant samples^21,24^ and h-FTAA visualised cellulose with surgical precision. This result was outstanding in lieu of the natural presence of lignin, a strong competitive binder of oligothiophene^1^ and hemicelluloses in the tissue. Our reported data further substantiates the effectiveness of oligothiophenes as a simple tool for non-destructive study and quality assessment of polysaccharides, in place of existing methods. With future development aimed to detect a wider range of polysaccharides, oligothiophenes are poised to become of great benefit to plant scientists, as well as in bio-refinery processes as real-time reporters of chemical reactions and feedstock compositions. As new low-cost/high-efficiency methods are developed and integrated into bio-refinery processes, societies endeavour to replace fossil materials by plant derived alternatives may be well within reach.

## Methods

### h-FTAA, glucans and plant tissues

Stock solutions (1 mg/ml) of h-FTAA in deionised water were stored at 4 °C. Microcrystalline cellulose fibres (M. cellulose, 100 μm) from cotton liners (CAS no. 9004-34-6), cellopentaose (CAS no. 2240-27-9), methyl cellulose (CAS no. 9004-67-5), 2-hydroxyethyl cellulose (CAS no. 9004-64-0), hydroxypropyl cellulose (CAS no. 9004-64-2), sodium carboxymethyl cellulose (CAS no. 9004-32-4) with a degree of substitution (DS) of 0.7 and 1.2 respectively, laminarin from *Laminaria digitata* (CAS no. 9008-22-4), starch from corn (CAS no. 9005-25-8), amylose from potato (CAS no. 9005-82-7), amylopectin from maize (CAS no. 9037-22-3), glycogen from bovine liver (CAS no. 9005-79-2) and dextran (CAS no. 9004-54-0) were from Sigma-Aldrich, Stockholm, Sweden. Celloheptaose and cellooctaose were from ElicityL, Grenoble, France. Glucans were dissolved/suspended in ‘ultrapure water’ of ‘type 1’ (ISO 3693). Polysaccharides were maintained as 2 mg/ml stock solutions at 4 °C, oligosaccharides as 1 mg/ml stock solutions in −20 °C. Brown onions (*Allium cepa* var. *cepa* – Linnaeus) and potatoes (*Solanum tuberosum* L) from local supermarkets were stored at 4 °C. Cellulose nanofibrils were prepared as previously described^1,25^. Oxidized pulp from bleached never-dried softwood pulp (Domsjö dissolving plus) containing 20% (w/w) dry content (~93% cellulose, ~5% hemicellulose) treated with 2,2,6,6-tetramethylpiperidine-1-oxyl radical (TEMPO) was defibrillated into a gel of nanofibrils dispersed in water by mixing, ultrasonification and mechanical stirring. Amorphous cellulose was prepared from pure, dry α-cellulose, kindly provided by Södra Cell AB, Sweden. α-cellulose (1.5 g) was first dried for 48 h at 100 °C and then mixed with 100 ml of dimethylacetamide (99.8%, Sigma-Aldrich, Stockholm, Sweden), containing 5% LiCl (≥99.0%, Sigma-Aldrich, Stockholm, Sweden). The mixture was heated to 80 °C for 1 h with constant stirring and then kept on a shaking board until a solution was obtained. The cellulose solution was then drop-wise precipitated into cold acetone or casted as a thin film onto a petri dish containing a thin layer of acetone which was removed as the film solidified.

### Optical recordings of h-FTAA – polysaccharide interactions

Aliquots (50 μl) of each oligo- and polysaccharide stock solution were pipetted into individual wells of a white 96-well round bottom microtiter plate (Corning, Stockholm, Sweden). The h-FTAA stock solution was diluted in PBS, pH 7.4, (Sigma-Aldrich, Stockholm, Sweden) to a final concentration of 1.33 μg/ml, from which 50 μl was added to each well. Samples were sealed with adhesive PCR plate seals (Thermo-Fischer, Stockholm, Sweden) and incubated at 4 °C on a rocking shaker for 30 min. The plate was then positioned in a Synergy MX platereader (Biotek, Stockholm, Sweden), the excitation spectrum of h-FTAA (emission at 600 nm) was collected between 300–580 nm and the emission spectrum (excitation at 500 nm) was collected between 520–750 nm. Spectra were collected with the instruments’ ‘top-down’ setting with 1 nm steps. Data from experiments performed in triplicates were processed and analysed using Prism 6 (Graphpad, USA).

### Staining of M. cellulose and plant tissues *in situ*

Slices (10 mm square, 1 mm thick) were cut from potatoes and brown onion scales. Onion epithelium was prepared by peeling off a thin, single-cell layer from the underside of a 10 mm square cut of the outermost peel of the onion. To prepare for fluorescence microscopy, tissue sections were incubated in wells of a 12-well plate (Sarstedt, Numbrecht, Germany) containing 2 ml h-FTAA (3.3 μg/ml) in PBS. Following 30 min incubation in room temperature, tissues were washed by immersion in PBS. Tissues incubated in PBS without h-FTAA served as negative control.

M. cellulose was stained by incubating a mix of 1 mg M. cellulose and 1 ml h-FTAA (3.3 μg/ml) in PBS for 30 min in room temperature. Unbound h-FTAA was removed by centrifugation (5000 g, 5 min) and the pellet was washed twice in PBS. Samples were mounted in a drop of mounting medium (DAKO, Stockholm, Sweden) on a microscopy slide, sealed with a glass coverslip, then analysed by multi-detector imaging, multi-laser/multi-detector analysis or spectral imaging.

### Multi-detector imaging

Multi-detector imaging represents a technique where emitted fluorescence from a sample exposed to one excitation wavelength is simultaneously detected by two or more photomultiplier tubes (PMTs). To enable this type of imaging, the confocal laser scanning microscope (Olympus, Stockholm, Sweden) was first loaded with the software Fluoview FV1000 (Olympus, Stockholm, Sweden) using factory pre-sets for Alexa Fluor 405, Alexa Fluor 488, Alexa Fluor 546 and Alexa Fluor 633. As this automatically loads the 405, 473, 559 and 635 lasers, bandwidth filters and PMTs into the ‘ON’ position, we manually adjusted or turned off these settings as required. By selecting Sequential acquisition by ‘line’ mode and placing the PMTs into ‘Group 1’, the resulting detection wavelengths were 430–455 nm, 490–540 nm, 575–620 nm and 655–755 nm. Unnecessary excitation wavelengths were then manually shut off in the ‘Acquisition settings’ panel. The same settings for Voltage, Gain and Offsets were applied to all detectors. Phase contrast images were collected in the transmitted light detector with excitation at 473 nm. Image stacks were collected using the lenses UPLSAPO 20 × NA0.75, UPLSAPO 40 × 2 NA0.95 and UPLSAPO 60XW NA1.2 (Olympus, Stockholm, Sweden).

### Multi-laser/multi-detector analysis

In the multi-laser/multi-detector analysis, a fluorophore is excited at multiple wavelengths, while emitted fluorescence is simultaneously detected by two or more photomultiplier tubes (PMTs). We excited h-FTAA at 405 nm, 473 nm, 535 nm and 635 nm. Our confocal laser scanning microscope (Olympus, Stockholm, Sweden) was first loaded with the software Fluoview FV1000 (Olympus, Stockholm, Sweden) using factory pre-sets for Alexa Fluor 405, Alexa Fluor 488, Alexa Fluor 546 and Alexa Fluor 633 and individually placed into separate ‘Groups’. Sequential acquisition in ‘Frame’ mode was selected during imaging. The resulting paired laser/PMT wavelengths were 405/430–450 nm, 473/490–540 nm, 535/575–620 nm and 635/655–755 nm. The standardised Voltage, Gain and Offset settings were replaced by individual calibration of PMTs using the ‘High-Low’ setting to avoid pixel saturation or cut-off of emitted fluorescence. Unstained controls were imaged using the same settings as used for their corresponding h-FTAA stained samples. Phase contrast images were collected in the transmitted light detector with excitation at 473 nm. Image stacks were collected using the lenses UPLSAPO 20 × NA0.75, UPLSAPO 40 × 2 NA0.95 and UPLSAPO 60XW NA1.2 (Olympus, Stockholm, Sweden).

### Spectral imaging and analysis

Emission spectra were collected on a Leica DM6000 B fluorescence microscope (Leica, Wetzlar Germany) equipped with a SpectraCube module (Applied Spectral Imaging, Israel). Laser/Bandpass filters 436/441–800 nm were used for h-FTAA. The emission profile of 4 pixels was obtained for each region of interest and presented using Prism 6 (Graphpad, California, USA).

### Preparation and analysis of images and videos

Fiji (Wisconsin, USA) was used to process image stacks into single optical projections, Z-projections, 3D projections and videos.

### Data availability

The authors declare that data supporting the findings of this study are available within the paper and its supplementary information.

## Electronic supplementary material

Supplementary Video 01

Supplementary Video 02

Supplementary Information
