# Supplementary material for: Stereochemical identification of glucans by oligothiophenes enables cellulose anatomical mapping in plant tissues
Source: Sci Rep. 2018 Feb 15;8:3108. doi: 10.1038/s41598-018-21466-y (PMC5814555; doi:10.1038/s41598-018-21466-y)
Supplement: Supplementary file 3 — Supplementary Information [file 41598_2018_21466_MOESM3_ESM.pdf]

SUPPLEMENTARY INFORMATION

Stereochemical identification of glucans by oligothiophenes enables cellulose anatomical mapping in plant tissues

Ferdinand X. Choong[1], Marcus Bäck[2], Anette Schulz[1], K. Peter. R. Nilsson[2], Ulrica Edlund[3], Agneta Richter-Dahlfors[1*]

[1] Swedish Medical Nanoscience Center, Department of Neuroscience, Karolinska Institutet, Stockholm, SE-171 77, Sweden

[2] Department of Chemistry, IFM, Linköping University, Linköping, SE-581 83, Sweden

[3] Fibre and Polymer Technology, KTH Royal Institute of Technology, Stockholm, SE-100 44, Sweden

Table of Content page

Supplementary Figure 1	2
Supplementary Figure 2	3
Supplementary Figure 3	4
Supplementary Table 1	5
Supplementary Table 2	6 – 7
Supplementary Video Legend 1	8
Supplementary Video Legend 2	8

Supplementary Figure 1

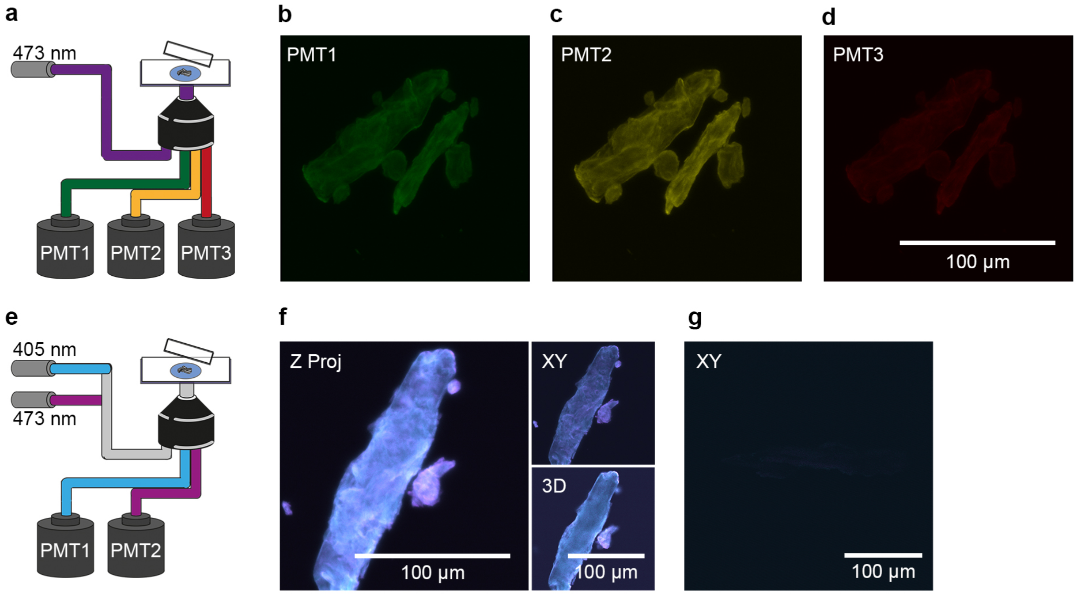

Multi-detector analysis of optical signals from h-FTAA bound to M. cellulose using confocal laser scanning microscopy

(a) Schematic showing the light paths for multi-detector imaging of h-FTAA bound to M. cellulose. A 473 nm laser is used for excitation. Emitted fluorescence from the sample is detected in three photomultiplier tubes (PMT) in the ranges 490 – 540 nm (PMT1, green), 575 – 620 nm (PMT2, yellow), and 655 – 755 nm (PMT3, red).
(b-d) Multi-detector analysis showing 3D brightest point projections of an image stack (24.96 µm stack, z-step = 0.64 µm) of h-FTAA stained M. cellulose excited with a 473 nm laser. Emitted fluorescence collected by **(b)** PMT1, 490 – 540 nm (green), **(c)** PMT2, 575 – 620 nm (yellow), and **(d)** PMT3, 655 – 755 nm (red) are shown. **(e)** Schematic showing the light paths for multi-laser/multi-detector analysis of h-FTAA bound to M. cellulose. The sample is exposed to sequential excitation with 473 nm and 559 nm lasers. Emission is detected frame-wise by PMT1 and PMT2 at 490 – 540 nm (cyan) and 575 – 620 nm (magenta), respectively. **(f-g)** Multi-laser/multi-detector analysis showing **(f)** a Z-projection (Z Proj), a single optical section (XY) at the centre of the crystal (optical plane number 12 of 26), and a 3D brightest point projection (3D) of an image stack (16 µm stack, z-step = 0.64 µm) of h-FTAA stained M. cellulose and **(g)** a single optical section (XY) at the centre of the crystal core of unstained M. cellulose.

Supplementary Figure 2

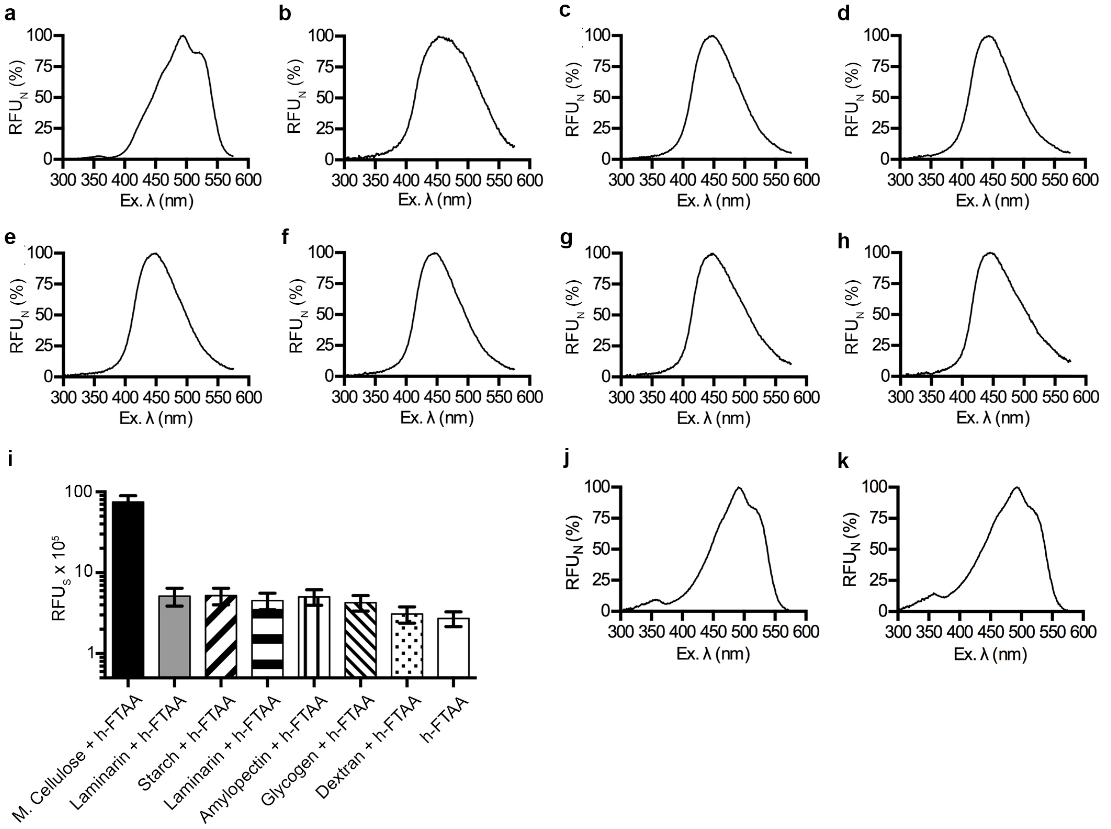

Optical signatures of h-FTAA interacting with α and β configurated glucans

(a-h) Normalised Spec-Plot of excitation spectra from h-FTAA interacting with **(a)** M. cellulose, **(b)** laminarin, **(c)** starch, **(d)** amylose, **(e)** amylopectin, **(f)** glycogen and **(g)** dextran, as well as from **(h)** h-FTAA in PBS, for easy determination of excitation λ_{max}, at peak fluorescence. RFU_N represents the emitted fluorescence at each excitation wavelength shown as percentage of the highest and lowest values in each spectrum. All spectra show average data complied from 3 independent experiments. **(i)** Emitted fluorescence (RFU_S) of h-FTAA interacting with the glucans shown in panels (a-g) when excited at 500 nm. h-FTAA with no glucans added is included as control. Average data and standard deviations from 3 independent experiments are shown. **(j, k)** Normalised Spec-Plot of excitation spectra from h-FTAA interacting with **(j)** cellulose nanofibrils, and **(k)** amorphous cellulose. Average data compiled from 3 independent experiments are shown.

Supplementary Figure 3

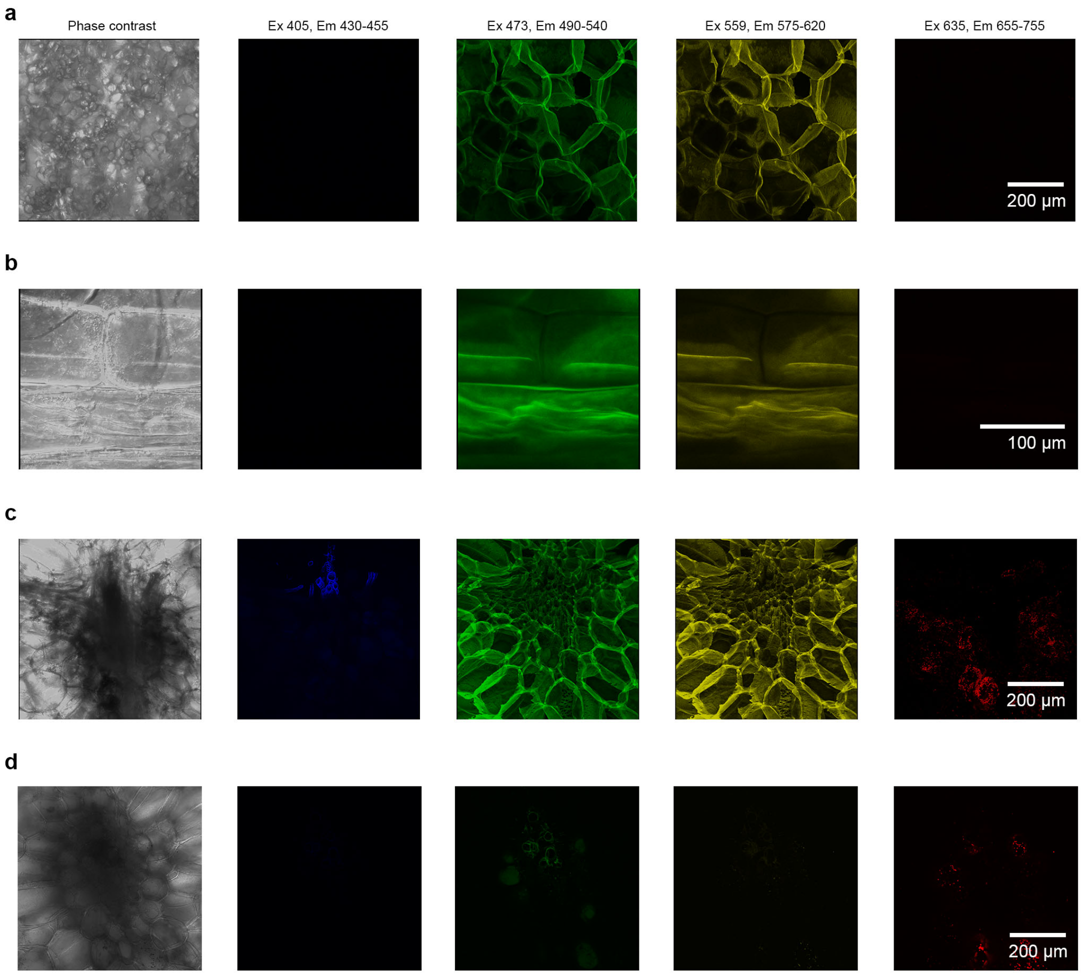

Multi-laser/multi-detector analysis of h-FTAA bound to plant tissues

Phase contrast (first column) and fluorescence microscopy performed by sequential excitation and frame-wise detection by PMTs at wavelengths indicated above each column (column 2-5). Fluorescence images show 3D brightest point projections of image stacks of **(a)** potato parenchyma (92.72 µm stack, z-step = 1.22 µm), **(b)** onion epithelium (38.64 µm stack, z-step = 0.56 µm), and **(c)** a cross-section of the vascular bundle in an onion scale (85.4 µm stack, z-step = 1.22 µm), all stained with h-FTAA. **(d)** Single optical section of cross-section of the vascular bundle in an onion scale without addition of h-FTAA.

Supplementary Table 1
Characteristics of cellulose, cellodextrins and cellulose derivatives

Trivial name	Appearance in D_2O	Carbohydrate composition [a]	R groups	Representative structural motif
Cellulose	Suspension	4-β-D-Glc*p*-(1→4)-β-D-Glc*p*	-	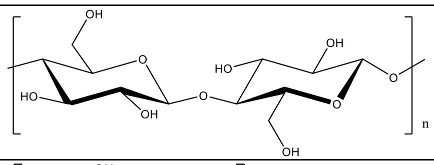
Cellopentaose	Homogenous solution	β-D-Glc*p*-(1→4)$_4$-β-D-Glc*p*	-	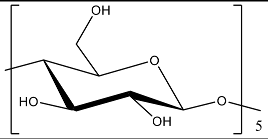
Celloheptaose	Homogenous solution	β-D-Glc*p*-(1→4)$_6$-β-D-Glc*p*	-	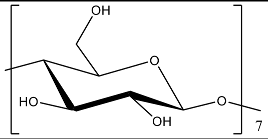
Cellooctaose	Homogenous solution	β-D-Glc*p*-(1→4)$_7$-β-D-Glc*p*	-	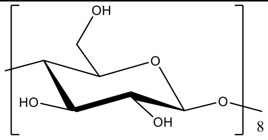
Hydroxyethyl cellulose	Homogenous solution	4-β-D-Glc*p*-(1→4)-β-D-Glc*p*	H or CH_2CH_2OH	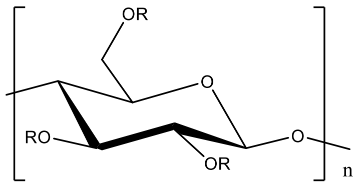
Hydroxypropyl cellulose	Homogenous solution	4-β-D-Glc*p*-(1→4)-β-D-Glc*p*	H, CH_3 or $CH_2CH(OH)CH_3$	
Methyl cellulose	Homogenous solution	4-β-D-Glc*p*-(1→4)-β-D-Glc*p*	H or CH_3	
Carboxymethyl cellulose, degree of substitution 0.7	Homogenous solution	4-β-D-Glc*p*-(1→4)-β-D-Glc*p*	H or CH_2COOH	
Carboxymethyl cellulose, degree of substitution 1.2	Homogenous solution	4-β-D-Glc*p*-(1→4)-β-D-Glc*p*	H or CH_2COOH	

[a] α/β is the configuration of the anomeric hydroxyl; arrows indicate covalent bond; Glc*p* = glucopyranosyl; numbers indicate the position of attachment to the next monosaccharide unit.

All structures are drawn with ChemDraw Professional 16, PerkinElmer Informatics.

Supplementary Table 2
Characteristics of analysed non-cellulosic glucans

Trivial name	Appearance in D_2O	Carbohydrate composition [a]	R groups	Representative structural motif
Laminarin	Homogenous solution	3-β-D-Glc*p*-(1→3)-β-D-Glc*p*-(1→6)-β-D-Glc*p*	-	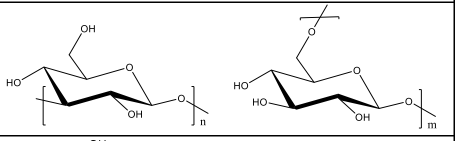
Amylose	Suspension	4-α-D-Glc*p*-(1→4)-α-D-Glc*p*	-	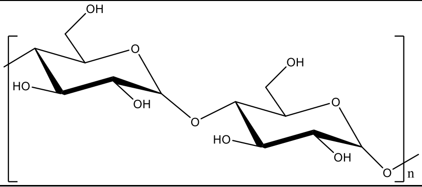
Amylopectin	Homogenous solution	4-α-D-Glc*p*<br>$\downarrow^{1}_{6}$<br>4-α-D-Glc*p*-(1→4)-α-D-Glc*p*	-	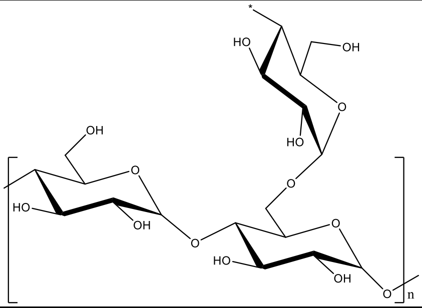
Starch	Cloudy suspension	Mixture of amylose and amylopectin	-	Mixture of amylose and amylopectin
Glycogen	Homogenous solution	4-α-D-Glc*p*<br>$\downarrow^{1}_{6}$<br>4-α-D-Glc*p*-(1→4)-α-D-Glc*p*<br><br>More branched than amylopectin	-	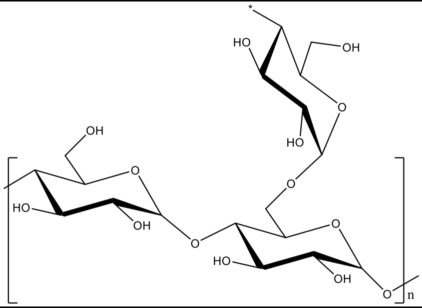
Dextran	Homogenous solution	6-α-D-Glc*p*<br>$\downarrow^{1}_{3}$<br>6-α-D-Glc*p*-(1→6)-α-D-Glc*p*<br><br>(1→2) and (1→4) branch points may also occur	-	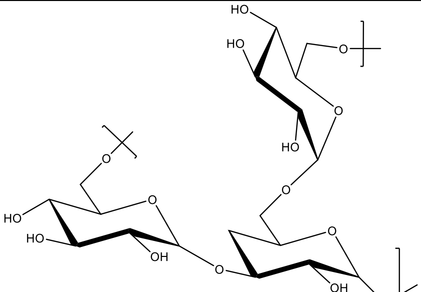
D-Glucose	Homogenous solution	Glc	-	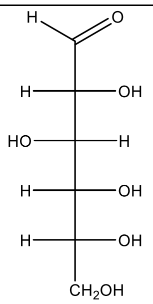

[a] α/β is the configuration of the anomeric hydroxyl; arrows indicate covalent bond; Glc*p* = glucopyranosyl; numbers indicate the position of attachment to the next monosaccharide unit.

All structures are drawn with ChemDraw Professional 16, PerkinElmer Informatics

Video Legends

Supplementary Video 1

Confocal microscopy of a cross-section of the vascular bundle in brown onion shows the cell wall thickening of the trachea, visualized by the intrinsic fluorescence of lignin (blue) winding through the tissue. By analyzing the 85.4 μm image stack as a video, the spiral type of trachea becomes apparent.
Scale bar = 100 μm

Supplementary Video 2

Video of h-FTAA stained onion tissue shows cellulose (green) anatomically located to the walls of cells in the vascular bundle. A video analysis of the 85.4 μm image stack generated by multi-laser/multi-detector analysis clearly delineates the spatial relationship between cellulose and intrinsically fluorescent chemical constituents and organelles. Chlorophyll inside the chloroplasts is shown in red, whereas lignin in the trachea is shown in blue. Structures within the cell wall, such as plasmodesmata and perforated sieve plates of the phloem, can also be observed.
Scale bar = 100 μm
